# A Smartphone App to Support Self-Management for People Living With Sjögren's Syndrome: Qualitative Co-Design Workshops

**DOI:** 10.2196/54172

**Published:** 2024-04-17

**Authors:** Claire McCallum, Miglena Campbell, John Vines, Tim Rapley, Jason Ellis, Vincent Deary, Katie Hackett

**Affiliations:** 1 Faculty of Engineering University of Bristol Bristol United Kingdom; 2 Institute for Collective Place Leadership Teesside University Middlesbrough United Kingdom; 3 School of Informatics University of Edinburgh Edinburgh United Kingdom; 4 Department of Social Work, Education and Community Wellbeing Northumbria University Newcastle upon Tyne United Kingdom; 5 Department of Psychology Northumbria University Newcastle upon Tyne United Kingdom

**Keywords:** self-management, mobile health, mHealth, eHealth, Sjögren's syndrome, patient participation, patient involvement, fatigue, chronic disease, focus groups, complex intervention development, mobile phone

## Abstract

**Background:**

Sjögren's syndrome (SS) is the second most common autoimmune rheumatic disease, and the range of symptoms includes fatigue, dryness, sleep disturbances, and pain. Smartphone apps may help deliver a variety of cognitive and behavioral techniques to support self-management in SS. However, app-based interventions must be carefully designed to promote engagement and motivate behavior change.

**Objective:**

We aimed to explore self-management approaches and challenges experienced by people living with SS and produce a corresponding set of design recommendations that inform the design of an engaging, motivating, and evidence-based self-management app for those living with SS.

**Methods:**

We conducted a series of 8 co-design workshops and an additional 3 interviews with participants who were unable to attend a workshop. These were audio recorded, transcribed, and initially thematically analyzed using an inductive approach. Then, the themes were mapped to the Self-Determination Theory domains of competency, autonomy, and relatedness.

**Results:**

Participants experienced a considerable demand in the daily work required in self-managing their SS. The condition demanded unrelenting, fluctuating, and unpredictable mental, physical, and social efforts. Participants used a wide variety of techniques to self-manage their symptoms; however, their sense of competency was undermined by the complexity and interconnected nature of their symptoms and affected by interactions with others. The daily contexts in which this labor was occurring revealed ample opportunities to use digital health aids. The lived experience of participants showed that the constructs of competency, autonomy, and relatedness existed in a complex equilibrium with each other. Sometimes, they were disrupted by tensions, whereas on other occasions, they worked together harmoniously.

**Conclusions:**

An SS self-management app needs to recognize the complexity and overlap of symptoms and the complexities of managing the condition in daily life. Identifying techniques that target several symptoms simultaneously may prevent users from becoming overwhelmed. Including techniques that support assertiveness and communication with others about the condition, its symptoms, and users’ limitations may support users in their interactions with others and improve engagement in symptom management strategies. For digital health aids (such as self-management apps) to provide meaningful support, they should be designed according to human needs such as competence, autonomy, and relatedness. However, the complexities among the 3 Self-Determination Theory constructs should be carefully considered, as they present both design difficulties and opportunities.

## Introduction

### Background

The need to improve the accessibility and quality of care for those with long-term conditions (LTCs) is an international priority [1]. In England alone, LTCs affect 15 million people [2] and account for 70% of health care spending [3]. Rheumatic diseases are LTCs with a particularly high prevalence in the United Kingdom and worldwide, having been estimated to affect up to one-fourth of Europeans [4,5] and a similar proportion of the population in the global south [6]. Sjögren's syndrome (SS) is thought to be the second most common autoimmune rheumatic disease [7] and is associated with poor quality of life [8] and high disease burden [9].

SS is a heterogeneous LTC, with a constellation of unpredictable and diverse symptoms [10,11]. A key characteristic of SS is mucosal dryness due to the destruction of exocrine (moisture-producing) glands by the body’s immune system, which particularly affects the eyes, mouth, and vagina [12]. In addition to dryness, common extraglandular features include persistent fatigue [13], chronic pain [14], sleep disturbances [15], and anxiety and depression [16]. People with SS report experiencing these symptoms as being interconnected, with the exacerbation of one symptom impacting others [17-19].

Similar to many other autoimmune diseases, SS does not have a cure [20]. Therefore, intervention efforts have focused on reducing the severity of symptoms; for instance, topical treatments are used for managing dryness [21]. Drug treatments for the systemic management of SS, such as hydroxychloroquine and rituximab, have had disappointing results in clinical trials [22,23]. Behavioral interventions that aim to improve the quality of life are a promising alternative; however, few interventions have been developed, and evaluations of their impact have been of low quality [21,24]. A recent stakeholder engagement study found that support for self-managing symptoms was a key priority for people with SS [25]. The term self-management has been defined as “the individual’s ability to manage the symptoms, treatment, physical and psychosocial consequences and lifestyle changes inherent in living with a chronic condition” [26,27]. To support the knowledge, behaviors, and attitudes required, self-management interventions should deliver a range of educational, behavioral, and cognitive techniques [28]. In SS, a targeted “complex” intervention is required, which delivers multiple techniques and targets multiple SS symptoms [29]. Our previous body of work with patients with SS found that they require different levels of support. Some require more complex individual support, but most people require lower levels of support with access to written information and digital self-management tools [29], which could be provided in the form of a website or smartphone app.

### Apps as a Support for Self-Management

SS shares multiple symptom and self-management similarities with other LTCs [30], including but not limited to neurological and autoimmune conditions such as rheumatoid arthritis, myalgic encephalomyelitis, and multiple sclerosis. Smartphone apps are a promising approach to support self-management of these LTCs [31,32] and other conditions such as type 2 diabetes [33], asthma [34], and hypertension [35]. Their increasing availability and functionalities enable complex intervention techniques to be delivered in the context of users’ daily lives when they are designed with consideration of users’ routines and choices [36]. User-centered design studies of LTCs have produced various app features and content [37] to support, for example, user education and cognitive strategies. However, app effectiveness can be limited by very low levels of user engagement [38,39]. Therefore, intervention developers must design apps that are more engaging and carefully consider how such engagement will ultimately lead to long-term behavior change [40]. For example, beyond simply providing information about how to perform techniques, apps can be designed to promote a sense of autonomy and motivation to engage in self-management behaviors over time [41,42].

To increase user engagement, apps should be user centered and person centered [43], that is, designed to fit within individuals’ current lives and daily activities [44]. People are more likely to use a new intervention if it can be incorporated into their existing habits, routines, and contexts [45,46]. Therefore, self-management interventions should account for and actively support how people manage their conditions currently [31,47]. Thus, to develop a useful, effective, and engaging app-based intervention that supports those with SS, there is a need to first understand their current self-management opportunities and challenges. To date, limited studies have only been conducted to understand the lived experience of symptoms [17,19,48] and have not explored the self-management of multiple diverse symptoms.

To gain an understanding of individuals’ self-management contexts, co-design and user-centered methods are useful [49]. These can involve practical design activities that elicit conversations regarding a topic of interest (such as self-management) to inform the development of a design, product, or intervention and have been used to develop digital health interventions [50,51]. Then, to understand how users in these contexts might best be supported in changing their self-management behavior, co-design findings can be interpreted using theories of motivation and behavior change [41].

Self-Determination Theory (SDT) [52] is one motivational theory widely used in interventions promoting health behavior change [53,54], including those for self-managing chronic illnesses [55,56]. SDT proposes that the constructs of competence, autonomy, and relatedness are required for individuals to be internally motivated to perform behaviors and sustain these changes over time. Situating qualitative findings within theoretical constructs facilitates the development of apps that are based on theory [42,57]. While intervention developers use SDT to inform their interventions, many do not explicitly link the theoretical constructs directly to their individual components, and we aimed to bridge this gap. To the best of our knowledge, there are no evidence-based, theory-driven, self-management apps for SS.

### Study Aims

We aimed to use an SDT framework to explore self-management challenges and approaches used by people with SS and to produce a set of design and therapeutic recommendations for a supportive and engaging app to aid self-management.

## Methods

The methods and subsequent results have been reported according to the COREQ (Consolidated Criteria for Reporting Qualitative Research) guidelines (Multimedia Appendix 1 [58]).

### Study Design

**Figure 1:**
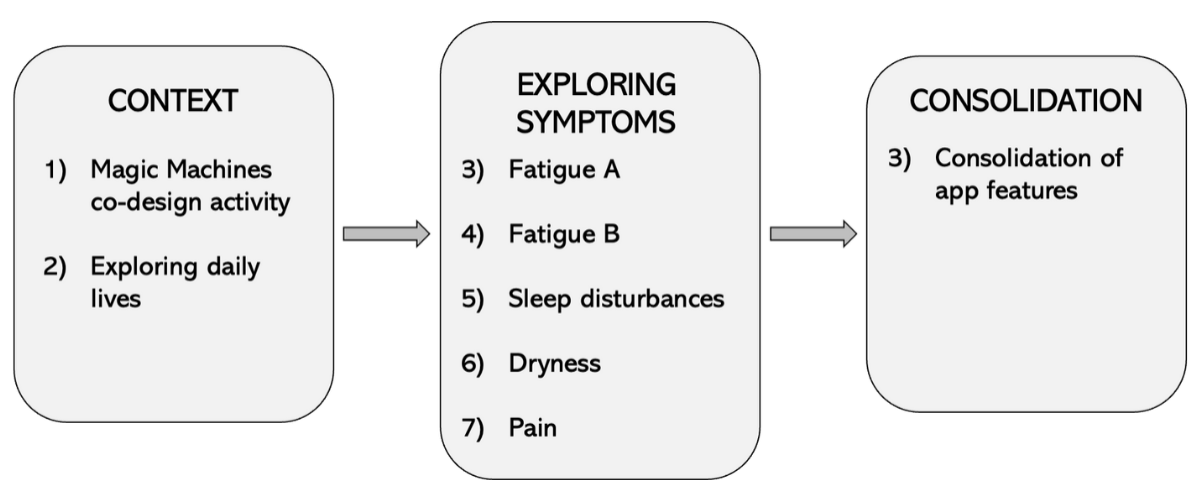
The procedural flow and topics of the 8 design workshops.

A consecutive series of 8 workshops with people living with SS was conducted over 7 months, each involving design activities and focused discussions (Figure 1). The first 2 workshops were open ended to broadly understand participants’ contexts (ie, key self-management challenges and overall self-management routines) and enable participants to become familiar with each other and to feel comfortable while discussing potentially sensitive and personal topics. We decided in advance to include a series of workshops, with each workshop dedicated to in-depth understanding of the self-management activities, challenges, and opportunities for each symptom. However, the order of symptom workshops and their exact discussion topics and activities were not predetermined; their sequential nature enabled us to iteratively design topics based on the findings from the previous session. For example, a clear theme emerged regarding symptom interrelatedness, so subsequent workshops included discussions about how participants managed interrelations among their symptoms. Furthermore, fatigue was a priority for all workshop participants (14/14, 100%), and therefore, 2 workshops were dedicated to this symptom.

Participants were given the option to attend ≥1 workshops. Several workshops were repeated to suit participants’ availability. To enable those who could not access any workshops due to other commitments and to include the experiences of younger people living with SS, 3 one-off semistructured interviews were conducted. These focused on the key self-management practices and challenges experienced by the respective participant.

### Ethical Considerations

This qualitative study received ethics approval from Northumbria University ethics committee (reference 11130). Informed consent was obtained before data collection, and travel costs were reimbursed.

### Recruitment

Workshop participants were purposively recruited from a regional UK SS support group (Northeast Sjögren's Syndrome Association). Advertisements were distributed via their member mailing list and Facebook page, and the research team presented the project at a support group meeting. The invitation was open to those diagnosed with SS by a physician, and potential participants were invited to attend as many workshops as they liked. Interested participants who were unable to attend due to their location or life commitments were invited to attend a video web-based interview. Additional participants were recruited via social media (a single tweet on Twitter [subsequently rebranded as X]) and invited to participate in the interviews only.

### Data Collection Activities

#### Overview

Workshops were conducted at Northumbria University, lasted approximately 90 minutes, and included a 10-minute comfort break. The interviews lasted 30 to 60 minutes and were conducted via telephone or videoconferencing software. Workshops were facilitated by 3 authors (CM, MC, and KH)—all were female postdoctoral (PhD) researchers trained in qualitative research methods and experienced in conducting qualitative research interviews and focus groups; one of them was also an occupational therapist (KH) with experience in SS symptom management. Several workshop participants (4/14, 29%) had attended clinics (conducted by KH), and 14% (2/14) had participated in previous studies (conducted by KH and VD). All participants (17/17, 100%) were briefed about the aims of the study. All workshops and interviews were audio recorded, and facilitators took field notes. In the following sections, we have outlined the focus of each workshop. The individual workshop topic guides are presented in Multimedia Appendix 2 [59-62].

#### Workshop 1: Magic Machines Co-Design Activity

This workshop introduced the series of workshops, included discussion about some key self-management issues experienced by participants, and involved a Magic Machines [54,59] craft activity where participants created some imaginative design solutions for another workshop participant. The Magic Machines activity aimed to elicit a broad range of knowledge about participants’ personal and technological needs through discussions about everyday problems related to their condition and potential solutions. Participants were asked to create an object, which addressed their partner’s daily challenge, using household objects and craft items. Data capture was focused on the conversations between participants about their “problem” while making their objects (a potential “solution”) and when describing their object to the main group at the end of the session.

#### Workshop 2: Exploring Daily Lives

The second workshop explored individuals’ “daily lives” and the self-management of symptoms. The discussion about daily lives invited participants to discuss their “typical day” in managing SS (ie, their habits and routines), how SS self-management was incorporated into their routines, and any related challenges that they experienced.

#### Workshops 3 to 7: Exploring Symptoms

These workshops explored the self-management of specific symptoms and their interrelationships through group discussions and invited participants to engage in basic sketching to articulate their self-management experiences and challenges. We preselected the symptoms for discussion based on our previous study where patients identified them as being important and impacting their daily activities [25].

#### Workshop 8: Consolidation

Sketching was used to explore how an app might be structured to support symptom interconnectedness and complexity. This design activity also elicited discussion about user experience and usability issues. All participants (5/5, 100%) attending this workshop engaged in sketching, but if time was insufficient, they were encouraged to further develop their ideas by articulating them verbally.

#### Interviews

Following the workshops, 3 semistructured, web-based interviews were conducted by CM. The interviews followed a schedule of open-ended questions to allow for flexibility (Multimedia Appendix 3).

### Data Analysis

Audio data were transcribed verbatim, pseudonymized, and combined into a corpus for analysis using NVivo (version 12; QSR International). Analysis was conducted in 2 phases using a hybrid approach, incorporating both inductive and deductive methodologies, to harness the advantages of both methods [63]. First, an inductive thematic analysis approach [64] was used, where 2 researchers (CM and MC) independently coded the data, generating an initial set of codes related to participants’ self-management perceptions and experiences. Then, these codes were applied and refined through the arrival of each new transcript, and independent coding was subsequently conducted by CM. Discussions during regular research team meetings (with CM, MC, and KH) related to the codes and their connections, importance, and relevance were conducted to group codes into themes.

Then, these inductive themes were mapped to the 3 SDT [65] constructs of competency (the sense of capability to perform activities and tasks), autonomy (experience of having control and choice over one’s actions and decisions), and relatedness (feeling of connection and belonging and meaningful interaction with others) by CM. SDT was chosen over other motivational theories because it emphasizes social context as a key factor in helping or hindering motivation, which matched a prominent theme in our inductive thematic analysis of social relations, along with other major themes we found related to empowerment, autonomy, and capability (or “competency” in SDT). The theory is also highly translational, enabling findings to inform intervention design [66]. Regular research team meetings were conducted to review and reach consensus regarding the categorization of themes based on the SDT constructs. Opportunities to support participants’ challenges associated with these themes through an app were also identified through discussions. Methodological rigor and credibility of findings were pursued through development of a codebook, maintenance of ongoing reflexivity, peer debriefings, and data triangulation (from interviews, focus groups, and observations during workshop activities).

## Results

### Participants

In total, 17 people with SS participated in the workshops and interviews: 14 (82%) of the 17 participated in the workshops (13/14, 93% women and 1/14, 7% men) and 3 (18%) of the 17 participated in the web-based interviews (3/3, 100% women). Participants’ ages ranged from 33 to 76 (mean 56.5, SD 13.95) years, and 82% (14/17) of them had a diagnosis of primary SS. The remaining 18% (3/17) of the participants had a diagnosis of secondary SS. The mean number of years since diagnosis was 7.5 (SD 7.88) years. Regarding employment status, of the 17 participants, 8 (47%) were retired, 6 (35%) were working full time, 1 (6%) was in part-time employment, and 2 (12%) were not working currently. All workshop participants (14/14, 100%) had links to a local SS support group in the north of England. Of the 3 interviewees, 1 (33%) was part of the same support group, and the remaining 2 (67%) were from Spain and Canada, respectively (both were aged <35 years). Workshop group numbers ranged from 2 to 7 participants and 2 to 3 facilitators in each session. Some participants attended some sessions and not others, whereas a “core” group of 5 participants attended most sessions. One participant (1/14, 7%) attended only the first workshop; others attended at least 2 sessions.

### Overview

Participants engaged in a wide range of self-management behaviors, including using prescribed and over-the-counter medications and treatments (ie, applying eye drops and gels; bathing and massaging the eyes; using humidifiers, skin creams, and vaginal lubricants; following mouth care routines; using pain medication; and using hot and cold compresses). They also used cognitive and behavioral techniques including activity pacing, goal setting, general exercise, relaxation, mindfulness, distraction, napping, sleep management and wind-down routines, and social support. Participants used various tools to support and facilitate the learning, use, and practice of these techniques, including books (eg, about managing fatigue), diaries (paper based and digital), websites and forums (eg, National Health Service or SS associations as both knowledge resources and social support), apps on smartphones and tablets (such as for yoga, breathing exercises, and mindfulness), wearables (to track physical activity), and other devices (eg, for relaxing music, “mindless” television, or distracting podcasts and comedy). Not all participants owned or used smartphones. Tools were used in addition to visiting friends and holistic wellness centers (eg, spas and mindfulness classes) and learning self-care techniques directly from health care professionals (eg, when to apply eye drops and more complex techniques such as activity pacing and graded exercise).

In the following sections, we have described the challenges that participants faced in managing their condition and their psychosocial needs in terms of competency, autonomy, and relatedness.

### Competency

Participants varied in the extent to which they felt competent and successful in self-managing their SS, and this was related to how well they had established a self-care routine. One participant had a very *“*strict regime,*”* which they felt was required to maintain their level of functioning. While hearing about such self-management strategies from others, Jim reflected about his competencies:

I’ve still got quite a lot to learn...although it has been a few years now, I think I still haven’t got a good routine...I listen to your explanation [of another participant’s routine] and I think, why can’t I get myself like that? I’m supposed to be Mr Organised. I am known as that in my life. My working life and my own home life. Yet with this, I have not gotten organised yet.

Jim

Regardless of whether participants had routines or described habitual self-management behaviors, their sense of competency in self-managing SS was still impeded by the complex nature of their symptoms. Isolating and targeting individual symptoms was not only perceived to be difficult to perform (*“*You can’t separate the different symptoms*”* [Jim]) but were also sometimes unhelpful, as it did not account for their accumulative negative impact:

It is the overall effect to me. That three [symptoms] I can cope with and then the next day one raises its head and floors me...That straw that broke the camel’s back effect, you know. [Patricia] 

Several participants believed that they could better manage their symptoms through self-management techniques capable of improving multiple symptoms simultaneously. Some had discovered these types of techniques accidentally. For example, participants recounted noticing, with surprise, that eye drops had helped not only their dryness but also mental and physical fatigue. Other participants purposefully sought and regularly used techniques that targeted multiple symptoms simultaneously. Mindfulness and relaxation techniques provided a sense of control and the ability to “keep a cap on” multiple symptoms before they became very severe. Others agreed that seeking these techniques was worthwhile if they resulted in minor improvements across multiple symptoms. Despite valuing self-management techniques that targeted multiple symptoms, most did not feel confident or knowledgeable about which techniques were beneficial.

Another challenge to participants’ sense of competency is how SS symptoms are not static but change over time. Participants described instances where individual symptoms would rapidly fluctuate in severity:

[They] come and go...one day you might have a headache, the next day you don’t. [Jim] 

Participants also explained experiencing longer periods where multiple symptoms were severe (described using phrases such as a “flare,” “phase,” or “wave”) or individual symptoms persisted (such as “a dry patch”). While, sometimes, the onset of symptoms appeared “gradually,” at other times, they changed rapidly, leaving participants feeling unprepared (“a phase hits you”). Fatigue and pain were felt to be particularly volatile and could become severe with no warning and “like somebody just switched a switch” (Penny). 

Participants varied in how they managed such changing symptoms. Many attended to symptoms as they arose or increased in severity on a moment-to-moment basis (ie, an adaptive or reactive approach). However, this often meant devising complex and intricate strategies and sequences to manage the new combination of symptoms experienced in that moment. For example, sleep disturbances that might be attributed to pain, dryness, or anxiety required participants to change their approach to getting back to sleep accordingly (“depending on how I am” [Penny] or “what problem I am having” [Jim]). Other participants seemed to disregard the changing combination of symptoms and addressed symptoms “one at a time” based on whether they felt successfully managed. For example, Julie noted the following:

I tend to find like I feel like my feet are sorted, so I am now sorting my eyes, so I’m kind of going through this list.

Addressing symptoms required constant adjustments for participants. Their variable nature meant that just at the point that the individual starts to feel in control of one symptom, a flare of another may occur. 

A final layer of complexity impacting participant competency was how symptoms often change due to environmental factors. For example, dryness was exacerbated by air conditioning, bright lighting, and other people’s aftershaves and perfumes, whereas navigating new and busy places could exacerbate mental fatigue. The unpredictable nature of environments outside the home made self-managing symptoms more challenging. While home was characterized as “familiar” and “unchanging,” participants felt that they needed to continuously estimate the potential impact of environments on their symptoms and plan accordingly:

You have to be very wary of where you’re going...you’ve got to be careful. I will not walk through [the shopping mall] in the perfumery because there is always somebody going to...pick up a bottle of perfume and [spray]...I go, oh my eyes! [Geraldine] 

This planning itself was exhausting to several participants, and it also meant that they lacked spontaneity in their lives*.* Participants also felt that symptoms were easier to manage at home because they could easily perform physical relaxation and self-care techniques when required, particularly during a flare. During such times Sarah remarked as follows:

I just don’t want to leave the house, I don’t want to do anything. I just want to go and have like 2-3 baths per day.

In contrast, when symptoms left them debilitated outside the house, participants had to adopt different self-management techniques such as soothing self-talk or be “rescued” by a taxi or friend. Overall, being outside the home meant that participants were less in control of their environments; had to continuously plan and predict how the environment might impact their symptoms, which was mentally and physically tiring; and had to use different techniques to suit different environments.

### Autonomy

Our analysis identified many examples of participants feeling that they had autonomy in the self-management of their condition; however, sometimes, the same factors that promoted autonomy also reduced confidence and competence. Participants believed that the availability of various techniques meant that they had options in their self-management; there were multiple “different ways” they could try to improve symptoms. The plurality of techniques appeared to provide reassurance that at least one would be likely to be effective:

I have six choices...I don’t beat myself up when it doesn’t work because I’ve already got something else in mind.

Patricia

This plurality and optimism could provide a strong drive to continue in their self-management activities.

Participants varied in how they kept track of different available techniques. One participant had self-help books at various locations in their home. Another participant explained that they had collated several techniques to create their own book:

I wrote myself a little book...[of] top tips...I just wrote maybe two dozen messages across the book at random, things that might give me a clue. [Debra] 

Other participants used an experimental approach:

It’s about learning...through trial and error...you’ll notice a pattern...you don’t know until you’ve done it for a few months.

Michelle

These were similarly characterized by the desire to try different techniques and to keep track of their effectiveness:

With time and experience you begin to realise what works and what doesn’t.

Penny

It was acknowledged that this required continuous effort and perseverance.

Having personal choice to decide which techniques to try, as opposed to being directed by a health care professional, provided some participants with a sense of control. Debra likened creating her book of techniques to developing a tailored smartphone app:

It is basically my own app that I’ve written for myself...I didn’t feel like being ordered around by anybody else...I don’t necessarily follow it. If it’s inside my book, I think, well alright, maybe I’ll try something else...I’ve still got some kind of control over things. [Debra] 

Therefore, developing this herself meant that she did not feel obligated to try any 1 technique. Although participants appreciated having the autonomy to choose techniques in a personalized manner, the credibility of these techniques was also very important to them. Perceived credibility seemed to give them confidence to go ahead and try them. Some participants indicated that they understood the distinction between evidence-based information and hearsay:

I am pretty much someone who will try anything once if there’s some evidence to support its effectiveness...Some people suggest real outlandish things, like you hear it and you’re like, “okay!” I mean, I’m glad that it works for you, but I’m not really sold on trying that just yet.

Ellie

Participants felt that information about their condition or how to manage symptoms should be credible. For example, Jim explained that simply being presented with multiple self-management techniques and options, without a rationale for why they might be helpful, would not suit him. Others stated that knowing information sources was *“*useful...[for] controlling symptoms and trying to minimise [them]*”* (Edith). Information from websites such as the UK National Health Service or regional and national SS organizations was deemed trustworthy.

Although participants respected expert advice and implemented it in their self-management, expert authority was often only 1 element in an autonomous process of symptom management decision-making. For instance, when faced with a conflict between their preferred routines and expert advice, participants trusted their own expertise and experience. Jim outlined how he fell asleep with the help of music or old comedy shows and that he would simply “ignore” any potential prompts about adjusting his bedtime routine if it meant removing his music from the bed (as may be advised as part of a sleep intervention).

For some participants, smartphones and associated apps appeared to contribute to feelings of autonomy regarding their self-management of SS. Those who used a smartphone reported using basic note apps to track symptoms or calendars built into the operating system to track feelings of fatigue. Experienced smartphone users described how their ubiquitous nature enabled quick access to information and could give them access to techniques whenever and wherever needed, regardless of their location. In sessions where feedback was given about potential app designs, participants expressed the value they would see in new apps that brought various techniques together, provided reminders to apply eye drops, and helped track symptoms in a simpler manner. For instance, Julie suggested that “a tracker or a journal...or something like that on the app would be helpful” as this could help her manage her forgetfulness, which she referred to as “brain fog.”

However, while smartphones could enable autonomy, they also posed challenges that could impact the users’ SS symptoms. It was also noted that looking at the screen of a computer or smartphone for a very long time could exacerbate eye dryness:

It’s okay [when it’s] short, but you can’t spend a long time looking at the screen, because your eyes are just too sore.

Mel

To overcome this, participants used their smartphones differently. Some described deliberately limiting the amount of time they used them in 1 session, and others described changing their device settings to increase the font size or darken the screens. In addition, participants mentioned improving on-screen accessibility to reduce their eye strain and listening to audio instead of reading text. Patricia, noted that when “I am having my brain fog*”* the complexity of most apps “would blow my mind.” Among participants, there was a sense that smartphone use was closely related to experiences of mental fatigue from their SS.

Overall, participants valued the diversity of SS self-management techniques that are available and experienced this as enhancing autonomy. Smartphones and both generic and SS-specific apps were viewed as an important part of this diversity and could provide in situ tailored support. However, the apparent abundance of techniques and availability of smartphones also posed a challenge to autonomy. Patricia recounted that soon after being diagnosed with SS, she was overwhelmed by the need to learn about multiple symptoms and techniques from many sources. However, for her, this felt similar to being “shot at” from multiple angles. Sometimes, the factors that enable autonomy can also constrain it.

### Relatedness

Relatedness refers to the manner in which participants operated in their social worlds and how their practices of managing SS were related to it. Participants explained that SS profoundly impacted their familial interactions, friendships, and other forms of social contact. Participants enjoyed social activities and cherished positive relationships as a source of social support. Socializing and participating in activities with others provided a positive “distraction” from their symptoms. However, self-management tasks could impact their ability to socialize and interrupt the flow of conversations:

When in company if you are out and about and talking to people...You have to keep popping off to go and put eyedrops in, in the loo.

Edith

Furthermore, engaging in certain social activities, such as going to the cinema with friends, required participants to perform additional self-care, for example, applying eye drops more frequently, which could irritate the skin around the eyes. Geraldine explained that although she enjoyed going to live theatre performances, she was now reluctant to go based on previously being “crucified” by a smoke machine.

Pacing was a helpful technique to manage fatigue, but it was not always received well by others in social situations and workplaces. Patricia recounted that she had been regarded as “selfish” by family and friends for cancelling plans while trying to manage her energy and fatigue levels. She also recognized that having to “book” people into her diary well in advance to support her planning and pacing efforts “frightens some people off.” Edith recalled that the need to take more breaks meant that she had to decide to leave her walking group as she was no longer able to keep up with her friends. In turn, this negatively impacted her feeling of belonging.

Communication was key while managing illness demands and relationships. Some participants created their own SS information sheets to give to friends and health care professionals. Creating opportunities to explain difficulties was conducive to receiving valuable social support. Penny’s husband had delegated several household tasks to her, which were conflicting with her pacing technique. Penny explained that after discussing the issue with him, he subsequently understood the need to balance activities and that they were able to do this together. Ellie noted the following:

I do think that it is helpful to have people that you can talk to about Sjögren’s. I mean I have a very close relationship with my family, and I have close friends who I do feel like I can confide in, and that is really helpful for me.

Ellie

The freedom and ability to be open and honest about their SS symptoms with trustworthy family members and friends were central to well-being and helped with symptom self-management. However, despite all efforts to communicate effectively, many participants believed that, often, family, friends, and even health care professionals did not fully understand SS. They felt frustrated that symptoms were dismissed, normalized, or incorrectly attributed to other issues such as “getting old” or menopause. Dealing with invisible, ever-changing symptoms was difficult. Multiple general practitioner visits with complaints about seemingly benign symptoms such as fatigue and thirst were sometimes received with skepticism, and the transient nature of these symptoms made the situation worse:

Then you’re fine and you think, “they’ll think I am putting it on.”

Geraldine

Any respite from symptoms made some participants worry that those around them would not believe them the rest of the time. Carol knew that relative to other conditions that may have 1 visible “major” symptom, her multiple symptoms were unlikely to garner support and understanding because of “Sjögren’s [and] all the little things that it has” (Carol). Some participants had stopped attempting to explain their symptoms to family and friends, saying that some symptoms were *“*very difficult for you to articulate...to somebody who doesn’t feel it” (Joan). This was particularly detrimental to relationships with health care professionals. When health care professionals seemed uncompassionate about their symptoms, some participants talked about “shutting down” and making a choice to no longer discuss their SS in consultations. This had negative consequences on participants who ended up feeling rejected and disengaged, and there was a perception that, sometimes, health care practitioners were not even aware of this relational and motivational shift. 

When participants felt disbelieved, it led to experiences of self-doubt. Ellie said she was “bounced around like 4-5 practitioners” to the point where she questioned her illness “almost as if it is in your head.” Carol resorted to maintaining an activity diary, in part to monitor her fatigue and to preserve her sanity. For her, the diary data provided a sense of external objectivity and an opportunity to feel validated when being questioned by other people:

By doing the [diary] you think, yes...I’ve got a problem and that graph tells me...it is a physical thing, it’s not in my mind.

Carol

Being diagnosed with SS was a lonely experience for some participants due to the challenges of family, friends, colleagues, and health professionals not relating to participants’ symptoms or condition. Social isolation was particularly pronounced for a younger person with SS:

I don’t know anyone else who has it. So, it is kind of isolating...I also had a hard time finding people who are...my age. So, I mean, I would definitely be interested in meeting younger women who are working, who are finding strategies.

Ellie

Overall, connecting with others with SS was important, and participants sought opportunities to meet others with SS, learn, and find the validation and understanding they did not receive from others without SS. Some joined support groups and attended scientific conferences to expand their social circle with other people with SS.

However, not all social contact with others with SS was deemed helpful:

Some of the interactions I had honestly more scared me than helped me because it was people who were really in the throes of severe illness and some who weren’t coping well, and it was sort of anxiety-provoking.

Ellie

Therefore, support from others with SS was generally more welcome when it was helpful and positive, as interactions with those who were struggling to cope could have a negative impact on participants.

Within the construct of relatedness, even positive self-management was found to impact social activity, but having highly supportive friends and family could mitigate this to some extent. Describing and explaining the various, ever-changing symptoms to colleagues, friends, and health professionals who did not fully understand the condition or symptoms could be particularly challenging, but external resources such as using diary data could be a helpful tool to aid communication.

## Discussion

### Principal Findings

We sought to understand the current self-management approaches used by people with SS to inform the therapeutic ingredients and design recommendations for a self-management smartphone intervention. To date, most studies of lived experience with SS have focused on how specific symptoms are experienced [17,18,48]. To the best of our knowledge, no studies have explored how people with SS perform the day-to-day work of managing their condition and navigating challenges as they do so. This is an important consideration when designing interventions, as those that draw upon users’ expertise are more likely to be used [38]. Therefore, we analyzed qualitative data collated through a series of workshops and interviews with people with SS inductively before mapping the themes to the 3 constructs of SDT (competency, autonomy, and relatedness) [52]. This theory was used because it can help identify the psychosocial and practical requirements to support autonomous motivation to adopt and sustain healthy behaviors and to improve well-being in a population [52,67]. Our findings were consistent with what Cartner [68] first described in her qualitative study with participants with SS: the *labor* of living with SS. For her and our participants, competency was an ongoing effort, never a completed achievement. The complex, multisymptomatic, volatile, and unpredictable nature of the condition meant that their hard-earned expertise was being constantly challenged. Having to adapt to an ever-changing and unpredictable challenge evokes the concept of stress, but more specifically, it is captured by the notion of *allostasis:* the work that needs to be performed to find stability within a situation that is constantly changing. When allostasis is frequent or continuous, more work needs to be performed, and our emotional, cognitive, and biological resources can become dysregulated. This is known as *allostatic load*—the psychophysiological wear and tear that occurs to a system that is constantly having to adapt—which has clear links to anxiety, depression, morbidity, and mortality [69].

The labor of the participants and its costs were also evident in the SDT domain of autonomy. Often, there was a degree of forced autonomy, with participants having to perform the epistemic labor of determining how to manage their condition for themselves. This involved ongoing research and even compiling their own resources. Discernment and discrimination were required to determine what advice to trust and follow and how to balance that advice against their own experience. Although this process was enabling, it was also potentially disabling as the process of gathering and compiling information worsened some SS symptoms.

Finally, in the realm of relationships, managing SS requires significant social labor. Often, participants were required to manage the expectations, lack of understanding, skepticism, and disbelief of others, including health professionals, and these efforts were often only partially successful, leading to self-doubt, isolation, and lack of adequate care and support for their illness. This is not dissimilar to the experiences others face with other fatiguing LTCs such as stroke, fibromyalgia, multiple sclerosis, and ankylosing spondylitis [70].

### Design Recommendations

Multimedia Appendix 4 [52] summarizes our key findings, which have been mapped to the 3 constructs of SDT, with identified therapeutic approaches and design solutions for each. The findings within these SDT domains were identified as targets for intervention by the participants. In the following sections, we have reviewed these domains and suggested what interventions might help and how the interventions could be incorporated into an app to support self-management.

A key finding within the competency domain was that SS was multisymptomatic, volatile, and unpredictable. Participants were keen for interventions that would impact >1 symptom at a time. A previous study that investigated patient strategies for self-management of inflammatory bowel disease had similar findings [71]. Several treatment approaches and their components discussed during the workshops could potentially address several symptoms simultaneously. For example, activity and sleep management strategies such as pacing and reflective activity diaries have been used to support self-management of pain, sleep disturbances, and fatigue [72-76], and previous studies that evaluated interventions targeting several symptoms have shown promising results. Therefore, we suggest that when designing complex interventions for LTCs, intervention developers should map the potential, identified intervention content to behaviors and symptoms and select techniques that target >1 symptom where possible, thereby placing a smaller demand on the user. While this may not always be possible, streamlining the intervention content where practicable is likely to decrease the possibility of becoming overwhelmed and thereby supports user competency.

The key challenge in the autonomy domain was the amount of work required by participants to determine how to manage their condition on their own. As with many other LTCs, a large part of the “burden of treatment” is shouldered by the person with the condition [77]. Our findings broadly indicate that technology-enabled symptom management could help with this work of illness management. Participants liked the idea of a smartphone app to support self-management. However, merely operationalizing technology is not sufficient to promote and support self-management. Güldenpfennig et al [78] found that poor design and well-meaning paternalism, for example, through automated support that takes active choice away from the user, may compromise autonomy and proactive self-management. Furthermore, intervention designers should aim to strike a more careful balance between the input of experts by experience and those of professional experts [25,78]. In our study, we found that people with SS managed their symptoms using different approaches but that all of them had arrived at their own set of strategies and management regimes through experience, research, and trial and error. Acknowledging the individuality of self-management and the necessity to experiment with different approaches would be a key part of any intervention. Having a repository of strategies in 1 centralized app, which would also allow them to add their own strategies, would seem to be a potentially useful resource. This aligns with previous studies of apps that provide resources while allowing customization and thus may support a user’s sense of autonomy [79-82] and move away from a top-down paternalistic or prescriptive approach to LTC management [83,84]. An app for SS would need to combine recognition of the labor of self-management while helping to support it in a manner that honors the user’s autonomy and existing wisdom, providing the ability to choose from a range of therapeutic content and to determine the order in which they interact with it.

The most difficult and often fruitless area of labor was observed within the relatedness domain. Participants were required to manage others’ expectations, lack of understanding, skepticism, and disbelief, often leading to a smaller social world, isolation, and difficulties in accessing help from health professionals. Again, any intervention needs to begin by acknowledging this labor and the emotional and social costs of having a poorly understood and invisible illness. Our findings also showed that there was often a tension between illness management and maintaining relationships. For example, it could become difficult to implement strategies such as pacing when others were involved, particularly when the person with SS had not fully disclosed their symptoms or condition to the people whom their self-management strategy may affect. Therefore, saying “no” could also be hard for participants, particularly when it was perceived that others would not understand. Other participants had found a solution by working on their means of communicating their difficulties with those around them. Winger et al [85] have found that greater practice of assertiveness and communication skills was associated with reduced pain interference and psychological distress in people with lung cancer, and assertiveness and communication is also a key component of an effective fatigue management intervention for people with rheumatoid arthritis [60]. Therefore, we recommend including assertiveness and communication strategies within a therapeutic self-management app for SS. When considering the design of the app, we recommend including some text to help the user provide a brief explanation about their condition, its symptoms, and their impact to share with health professionals, colleagues, or people in social settings, as needed. We also recommend designing opportunities to practice assertiveness and communication skills within the app for those who may find it helpful.

In summary, our findings suggest that some of the key areas of concern for participants were potentially addressable through an intervention. A common starting point for any approach should be an acknowledgment of the real costs and the daily hard work of having an unpredictable, volatile, and multisymptomatic LTC. Any therapeutic approach needs to be designed to help with this labor; to acknowledge the social, emotional, and physical costs of having and managing SS; and to appreciate the wisdom that the “end user” of the app or intervention will have already accumulated. Strategies obtained from Acceptance and Commitment Therapy [86] and Compassion Focused Therapy [87] could be useful as they have been used to target the psychosocial impact of other related health complaints such as chronic pain [88]. Next, specific strategies (eg, pacing and sleep management) that could help target multiple symptoms or single symptoms in sequence would be useful. Finally, support to perform some of the social labor involved in living with SS should be a key component. In Multimedia Appendix 4, we have further specified the areas of intervention and suggested the broad therapeutic approaches that might be useful.

Regarding our use of the SDT framework, while it was useful to structure our thinking about intervention development, we also noted that the constructs of SDT often existed in a state of tension with each other, where successfully fulfilling the requirements of one construct leads to reduced functioning in another. As noted previously, this tension occurred between competence and relatedness, where symptom management conflicted with maintaining social bonds. Similar tension existed between autonomy and competence, where participants struggled to feel competent if presented with several self-management options. The SDT states that all 3 fundamental needs have to be met for internally motivated, self-determined behavior to occur [52], but we tentatively suggest that the theory needs to consider moments when some needs stand in opposition to each other. Making the nature of these tensions explicit to the users of an intervention or app would be a key part of its opening narrative.

### Limitations

The extent of transferability of our findings to other LTCs is not yet known. However, studies of other autoimmune conditions have demonstrated the same need to self-manage complexity—people with inflammatory bowel disease reported that symptoms (pain, fatigue, and diarrhea) changed over time and could be interconnected at different times, and they required a highly individualized management strategy to “balance” the illness and attend to dynamic fluctuations in symptoms [71]. Overall, our findings may provide insight into how several other autoimmune conditions are self-managed or could be self-managed with the use of an app. However, owing to the nature of the complexity we captured in this study, transferability of our findings to other contexts may be limited. The 17 participants in this study included only 1 (6%) man, which may mean that any unique difficulties experienced by men with SS have been missed in our study. However, SS has a female-to-male incidence rate of 16:1 [89], and the gender makeup of our participants is representative of the wider SS population. Another limitation was that we did not formally collate information about smartphone ownership from participants. Such data should be collated in future similar studies. A final limitation is that most of this study was conducted within the United Kingdom, with only 67% (2/3) of the interviewees living outside the United Kingdom. Therefore, we cannot assume that similar findings would be replicated in other geographical contexts.

### Future Studies

Future studies should operationalize the findings of this study to construct an intervention protocol that could be implemented via a smartphone app for the management of SS and empirically optimize its content through pilot and feasibility testing. Furthermore, future studies may explore the transferability of our findings to the self-management contexts of other autoimmune and fluctuating conditions. Our target users were those with primary or secondary SS; future studies should consider how user age influences the design requirements in this patient group.

### Conclusions

In conclusion, therapeutic and design approaches for SS should be constructed in both bottom-up (ie, based on the self-management challenges that prospective users already experience) and top-down (according to the most effective treatments documented for SS) formats. For people with SS, choosing to involve an app in their self-management has the possibility of being counterproductive—by adding to their experience of fatigue and becoming overwhelmed. Therefore, the design of a self-management app for SS should support the user in performing the physical, cognitive, emotional, and social work of self-management and should be careful not to add to their already high self-management costs.

## Appendix

Multimedia Appendix 1 COREQ (Consolidated Criteria for Reporting Qualitative Research) checklist.

Multimedia Appendix 2 Workshop topic guide.

Multimedia Appendix 3 Interview schedule.

Multimedia Appendix 4 Key findings with potential therapeutic and design recommendations mapped to the Self-Determination Theory domains.

## Abbreviations

COREQ: Consolidated Criteria for Reporting Qualitative Research
LTC: long-term condition
SDT: Self-Determination Theory
SS: Sjögren's syndrome
