# Supplementary material for: A Smartphone App to Support Self-Management for People Living With Sjögren's Syndrome: Qualitative Co-Design Workshops
Source: JMIR Hum Factors. 2024 Apr 17;11:e54172. doi: 10.2196/54172 (PMC11063884; doi:10.2196/54172)
Supplement: Multimedia Appendix 2 [file humanfactors_v11i1e54172_app2.docx]

**Multimedia Appendix 2.** Workshop topic guide.

| **Workshop Topics** | **Overview** | **Breakdown of Activities** |
| --- | --- | --- |
| **1) Magic Machines Codesign Activity [59]** | To explore the context, understand how people with SS conceptualise their self-management and elicit some of the key self-management challenges they face, this workshop involved each group member creating a “magic machine” [59] for another group participant. Participants arranged themselves into pairs or threes and were invited to describe to each other experiences of their most severe symptoms, their self-management approaches, and any challenges they experienced in managing symptoms. Next, participants were invited to create a magic machine that could help their partner to manage their symptom/s, using a selection of craft materials. To encourage creativity and open discussion, participants were asked to think beyond what is physically possible when considering the functionality of their machine [59]. Participants worked for approximately 20 minutes to create machines for their partner, discuss whether the machines created met their needs, and iterate on the design, before presenting and discussing their machines in a full group. In this full group discussion, facilitators prompted participants to reflect on whether the machine would be useful to support their own self-management, and why or why not. | 1. **In pairs:** Magic machine [59] task. Participants tell each other about an aspect of their condition or a symptom which impacts on their life. Each participant creates a magic item out of the available craft materials for the other person that will support them with managing that problem/symptom. 2. **In the main group:** Each participant, demonstrates the object they made for their partner and explains its purpose. 3. **Group discussion:** explore initial user experience challenges for different components/ how the app might fit into their daily lives |
| **2) Exploring Daily Lives** | To further understand the context, this workshop explored individuals’ “daily lives”, and the self-management of specific symptoms. The daily lives discussion invited participants to discuss their ‘typical day’ in managing Sjogren’s (i.e. their habits and routines), how SS self-management was woven into these, and any related challenges experienced. Focussed discussions on specific symptoms (fatigue, sleep, pain and dryness) invited participants to discuss the impact of each symptom, both individually and in relation to each other. Participants were encouraged to discuss specific self-management techniques used for each symptom, including any technologies used, and the challenges in managing each symptom among others.  Any discussions of smartphones or apps were probed further to elicit discussions on challenges and opportunities using these. | 1. **Group discussion:** About a typical 24-hour day of living with SS, to understand social, physical, emotional context and challenges/roadblocks/barriers and facilitators to activities. Participants were invited to bring in photographs to illustrate aspects of a ‘typical’ day prior to this session and annotate them to help prompt this discussion. Participants were invited to discuss how each of their symptoms impacted on their daily activities and on each other. 2. **Group discussion:** About individuals’ own SS management strategies and own “fixes”; technology use and attitudes towards an app for self-management; social support; imbalances in 24-hour day (i.e. is anything missing due to SS?) |
| **3) Fatigue A – Self-management strategies and metaphors** | This workshop focused on participants’ individual experiences of self-managing their fatigue and involved discussion and feedback regarding approaches and strategies used to support people with fatigue within a local clinical service [61] and within a rheumatoid arthritis fatigue management programme [60]. Participants were also invited to sketch metaphors to describe how their fatigue felt as a way of explaining it to others. | 1. **Presentation by facilitator:** Of fatigue interventions/intervention components from a local National Health Service (NHS) CRESTA Fatigue Clinic [61] and RAFT study [60]. 2. **Group brainstorm:** To identify fatigue coping strategies used by focus group participants and to identify where participants locate their information and self-management support materials/tips/advice. 3. **Group discussion:** On suitability, acceptability, feasibility of fatigue strategies and RAFT [60] and CRESTA [61] techniques 4. **Sketching in pairs**: Sketch metaphors for fatigue. Transform a coping strategy from the brainstormed list into an app to generate ideas for the fatigue component of the app. 5. **Group discussion**: Prompted by the sketches drawn by participants. |
| **4) Fatigue B – Exploring apps** | In this workshop, participants explored in pairs several apps downloaded from the Apple App Store and Google Play, which could be used to help self-manage fatigue. | 1. **Exploration of existing fatigue management apps and small group think aloud discussions:** Participants divided into smaller groups with a facilitator to explore existing free apps put on devices handed to them to explore usability, existing app features and content and facilitated discussion on relevance, concerns, and usefulness. 2. **Group discussion:** To identify important fatigue management features which should be in a future app. |
| **5) Sleep Disturbances – Self-**  **management experiences and**  **exploring apps** | Participants were encouraged to shared experiences on sleep disturbances and tips for managing them. They were also invited to explore in pairs several apps downloaded from the Apple App Store and Google Play and Think Aloud [62] techniques were used to capture their thoughts. | 1. **Group discussion:** To identify sleep strategies used by participants and to identify where participants locate their sleep information and self-management support materials/tips/advice. 2. **Brief presentation by facilitator:** On cognitive behaviour therapy for insomnia (CBT-I) intervention and its components. 3. **Group discussion:** Acceptability of CBT-I components and strategies used by other participants for SS**.** 4. **Exploration of existing sleep/CBT-I apps and small group think aloud discussions:** Participants divided into smaller groups with a facilitator to explore existing free apps put on devices handed to them to explore usability, existing app features and content and facilitated discussion on relevance, concerns, and usefulness. |
| **6) DRYNESS – Self-management experiences and metaphors** | Participants were invited to discuss their experiences of their dryness symptoms and discuss techniques they used to self-manage these. | 1. **Group brainstorm:** To identify participants’ own strategies to manage pain and dryness management and to identify where they locate their information and self-management support materials/tips/advice. 2. **In pairs:** Sketching app ideas and metaphors for dryness to understand how to present information about dryness within the app. |
| **7) PAIN - Self-management experiences and metaphors** | Participants were invited to discuss their experiences of their pain symptoms and discuss techniques they used to self-manage these. | 1. **Group brainstorm:** To identify participants’ own strategies to manage pain management and to identify where they locate their information and self-management support materials/tips/advice. 2. **In pairs:** Sketching app ideas and metaphors for pain to understand how to present information about pain within the app. |
| **8) CONSOLIDATION OF APP FEATURES** | Sketching was used to explore how an app might be structured to support symptom interconnectedness and complexity. The research team presented initial sketches of what the app “home screen” may look like to prompt discussion. Participants were then asked to sketch their own app home screen and any other app screens they desired. This design activity also elicited discussion around user experience and usability issues. | 1. **Brief presentation by facilitator:** Demonstrating wireframes which include the overall app containing sleep and fatigue of mockup components of app at its current stage of development. 2. **Group discussion:** Feedback on app acceptability, potential usability. 3. **Group discussion:** How do multiple symptoms impact on each other? Discussion on how participants would like components work together in an app to help support multiple symptoms. 4. **Sketching activity:** Participants to draw a sketch of a SS self-management app home screen and to show how this would lead to different components within such an app. 5. **Group discussion:** On how final thoughts on any changes to the structure and content of the future app. |
