# Supplementary material for: A Smartphone App to Support Self-Management for People Living With Sjögren's Syndrome: Qualitative Co-Design Workshops
Source: JMIR Hum Factors. 2024 Apr 17;11:e54172. doi: 10.2196/54172 (PMC11063884; doi:10.2196/54172)
Supplement: Multimedia Appendix 3 [file humanfactors_v11i1e54172_app3.docx]

Multimedia Appendix 3. Interview Schedule

1. **Could you tell me a bit about you: your age, the country you live in, whether you’re currently working (and your occupation), when were you diagnosed?**
2. **We have been exploring the symptoms frequently experienced by people living with Sjogren’s syndrome, including dryness, fatigue, pain, sleep issues. Have you experienced any of these, and could you tell me a bit about that?**
   - Do you experience all of these every day, or do your symptoms change over time?
   - Some people have told us that these symptoms can be connected (e.g. one triggers another), do you also feel that way? If so, how have you experienced your symptoms being connected?
   - Is there anything else you have experienced, you think might be connected to Sjogren’s syndrome? (e.g. some people experience low mood).
   - If you’re happy to discuss it: what kinds of impact do your symptoms have, if any, on your life? (e.g. work, social, other).
3. **How do you currently manage your symptoms, if at all?**
4. **What are your experiences and perspectives on using apps / technology for helping manage Sjögren’s syndrome?**
   - Which apps and technologies, if any, do you use? When do you use these?
5. **What benefits and challenges might there be in using apps for managing different aspects of Sjögren’s syndrome?**
6. **What kinds of app features would be helpful to improve sleep, fatigue, chronic pain, dryness?**
   - Which features would be the most and least important, and why?
7. **What kinds of information would you like the app to include about Sjögren’s syndrome?**
8. **Do you have any questions for me?**
