# Supplementary material for: A Smartphone App to Support Self-Management for People Living With Sjögren's Syndrome: Qualitative Co-Design Workshops
Source: JMIR Hum Factors. 2024 Apr 17;11:e54172. doi: 10.2196/54172 (PMC11063884; doi:10.2196/54172)
Supplement: Multimedia Appendix 4 [file humanfactors_v11i1e54172_app4.docx]

**Multimedia Appendix 4.** Key findings with potential therapeutic and design recommendations mapped to the Self-Determination Theory domains.

| Self-Determination Theory construct* and categories | Current self-management practices and preferences | Self-management challenges | Therapeutic approaches and solutions | Potential design solutions |
| --- | --- | --- | --- | --- |
| **Competency** | | | | |
| Managing several symptoms | - To manage several connected symptoms, there is a preference for techniques that target >1 symptom simultaneously, where possible - Participants would like to know whether the interventions address symptoms individually and whether there is potential for a “knock-on” positive impact on other symptoms | - Lack of knowledge about techniques that can target multiple symptoms | - Include tips and tricks obtained from multiple people with SS^a^ - Emphasize that one-size-fits-all approach is not applicable here - Encourage users to experiment and trust their own experience - Prioritize the inclusion of evidence-based self-management techniques targeting >1 symptom, when possible - Provide a clear rationale for the inclusion of techniques and be explicit in how they aim to help with different symptoms or symptom combinations - Include self-help techniques that target individual symptoms, where there is no symptom crossover | - Focus the intervention narrative/user flow on techniques that target >1 symptom simultaneously, where possible - Use these core techniques to organize the intervention (ie, a “technique-led” information architecture, where, for example, the main tabs in an app are techniques) - Include a rationale before each technique |
| Rapidly changing symptoms | - To manage flares and rapidly changing symptoms, some adapted their strategies on a moment-to-moment basis and prioritized the most severe symptom - Others reported disregarding their current state and persisting with self-management strategies for individual symptoms until they felt sufficiently managed in the long term | - Symptoms are volatile and difficult to control or influence - Participants rarely believed that they had sufficiently addressed or managed one symptom before “moving on” to the next symptom | - Acknowledge the volatility of and difficulty of living with changing symptoms within the text/content - Encourage self-compassion and acceptance of work and costs of dealing with the condition (eg, using Acceptance and Commitment Therapy–informed content [68]) - Encourage users to identify valued activities that can be performed with symptoms - Include content to enhance self-efficacy and self-belief - Potential therapeutic techniques to support this may include the following: graded activity, encouraging beliefs about capabilities, clear statement of “you are not alone,” problem-solving, and goal setting | - Use design strategies to improve the sense of self-efficacy/sense of control/capability - Provide simple tools for tracking the therapeutic techniques used, perceived self-efficacy in enacting them, and associated symptom effects - Include community features that support the sharing of experiences specifically associated with the management of multiple symptoms |
| Effects of the changing environment | - Participants reported needing to regularly assess the impact of the environment on their symptoms every time it changed (eg, the air quality), particularly in new situations - They needed a strategy to minimize the impact of these environmental fluctuations | - Planning how to respond to various possibilities can be exhausting and cognitively taxing | - Include an action planning tool that supports users in breaking plans into smaller tasks with flexibility for adjustments as required | - Design an activity planning tool with a simplified design to prevent users from becoming overwhelmed - Integrate features that use open-source air/environmental quality data sets to help users plan activities outside the home, and use community tools to highlight potential “hot spots” for problematic spaces |
| **Autonomy** | | | | |
| Numerous options | - Having a multitude of self-management techniques to choose from provided a sense of optimism (there are other options to try) | - Having numerous potential self-management techniques to choose from was felt to be overwhelming | - Provide users with a streamlined toolkit of relevant and appropriate therapeutic techniques - Provide a suggested order to try these techniques (if this is warranted therapeutically), while not restricting the user’s autonomy to choose or prioritize the techniques - Promote the use of experimentation to try 1 technique at a time | - Simplify the interface/reduce the possibility of becoming overwhelmed through simple and minimalistic design - One option is to present techniques one at a time, while still allowing users to override the order (avoid funneling or a “tunneled” information architecture) - Help the user to track what has worked for them and discard, mute, or deprioritize those that have not or is not relevant to them |
| Independence and control | - Some valued independence/control over choosing the self-management techniques - Others wanted guidance regarding which techniques to use - Independent decision-making was supported by understanding the rationale for using a particular technique and the credibility of information | - No challenge was identified | - Provide a rationale for techniques, ensure that techniques are evidence based, list credible sources, and maintain them up to date - Provide guidance regarding the suggested techniques for those who require it | - Provide *optional* tailoring and guidance regarding techniques to try - Design the techniques as recommendations or options, which users can either “turn off” or select - Use design strategies that enhance a sense of credibility, for example, by providing links or references to credible sources that informed the content or using logos from sources (eg, NHS^b^) as appropriate - Provide community features that highlight the use of techniques by other users and perceived self-efficacy |
| Customizable app features | - Some used apps to self-manage and had created paper-based systems that they likened to an app - Participants reported several benefits of a proposed SS-focused app (ubiquity/ability to learn about techniques anywhere); the collated, easy-to-find information rather than disparate sources; potential to remind them about new techniques to try and demonstrate how to perform them; or time-specific/context-specific reminders for when to use particular techniques (eg, apply eye drops) - Beyond apps, the tactile features of wearables were appreciated, and audio features alongside text could potentially reduce the impact of eye-related symptoms | - Looking at the screen of a computer or smartphone for a very long time could exacerbate eye dryness and mental fatigue | - Provide reminders to limit the length of sessions on the smartphone and take breaks from using technology | - Present the information in a “bite-sized” manner (not multiple menus/pages of text) - Design the app to be compatible with the accessibility options users already implement, including increased font size, ability to darken the screens, option to listen to an audio recording of the in-app text, and use of tactile vibrations to convey information - Design apps that work across an ecosystem of connected devices (eg, smart watches for reminders) and consider other forms of multimodal interaction to reduce screen time (eg, emphasize audio-based and vibration-based reminders) |
| **Relatedness** | | | | |
| Socializing | - Participants felt that socializing with others provided a positive “distraction” from symptoms | - Symptoms and effective self-management (eg, pacing and prioritizing) limited participants from socializing and performing activities with others | - Plan, prioritize, and pace social contact as part of an activity management strategy - Treat social activities similar to other activities, and encourage users to have a balance of different types of activities within their week - Arrange for close friends or family to occasionally act as “ambassadors” at agreed times when appropriate, to explain about the impact of the condition to others and decrease the communication burden | - Include reminders for users to enter social events in a planning tool |
| Communicating with other people | - Explaining symptoms and self-management strategies to other people sometimes resulted in them being more understanding and accommodating - Some participants found ways to self-manage and structure their days that enabled them to continue their social life - Carrying a paper document that explained the condition and symptoms was a useful way of communicating with others, including HCPs^c^ - Some people used activity diaries to demonstrate the impact of SS on their daily lives to others | - Users may struggle to communicate their condition, symptoms, needs, and therapeutic decisions to others, which impacts their relationships and their ability to self-manage well - Explaining the condition and its symptoms to others needs effort - Other people sometimes seem to lack understanding of the invisible condition, which can lead to feelings of frustration and self-doubt for the person with SS | - Provide assertiveness techniques to users, with which they can convey their needs to others without undue anxiety - Provide access to credible sources of information that can be used to demonstrate the credibility and validity of their symptoms when these are challenged - For those who struggle to communicate their needs, provide a written, customizable template for communicating with others (particularly in new settings) | - Include examples and exercises that train users in assertiveness and communication techniques - Include text such as preprepared communication cards for various audiences (employers, HCPs, work colleagues, etc) to aid communication about the condition; this should speak to others on behalf of the user to succinctly explain the condition, symptoms, and their impact - Include visual summaries of completed activity diaries to easily convey information to the user and others - Consider apps that are designed for spouses and family carers to support shared understanding of the condition and to distribute the social labor of advocating for oneself to friends and relations |
| Other people with SS | - Participants described wanting to meet others with SS who understood what it was like to live with SS - Participants accessed or wanted to access an SS forum - Speaking with role models could provide comfort | - Not knowing anyone similar to them with SS felt isolating, and it did not feel easy to identify with celebrities who had SS - However, others who have SS could report negative experiences, which may result in increased anxiety or frustration - Going outside the home required planning for any potential environmental impact on symptoms; going out to meet others with SS could be difficult | - Encourage through the use of others’ stories - Make it clear that each person has their path and everyone struggles sometimes | - Include examples and quotes from real people with SS - Use validating language - Use persuasive language to facilitate self-efficacy - Include a feature that asks users to recall an incident of overcoming a difficult situation - Provide community features that connect people with SS via simple asynchronous and synchronous (live and real-time) chat and audio conversations - Provide guidance for setting up internet-based social visits for people with SS |

^*^According to the Self-Determination Theory [52].

^a^SS: Sjögren’s syndrome

^b^NHS: National Health Service

^c^HCP: health care professional
